# Protective Effect of *Boerhaavia diffusa* L. against Mitochondrial Dysfunction in Angiotensin II Induced Hypertrophy in H9c2 Cardiomyoblast Cells

**DOI:** 10.1371/journal.pone.0096220

**Published:** 2014-04-30

**Authors:** Ayyappan Prathapan, Vadavanath Prabhakaran Vineetha, Kozhiparambil Gopalan Raghu

**Affiliations:** Agroprocessing and Natural Products Division, CSIR-National Institute for Interdisciplinary Science and Technology (NIIST), Thiruvananthapuram, Kerala, India; Instituto Nacional de Cardiologia I. Ch., Mexico

## Abstract

Mitochondrial dysfunction plays a critical role in the development of cardiac hypertrophy and heart failure. So mitochondria are emerging as one of the important druggable targets in the management of cardiac hypertrophy and other associated complications. In the present study, effects of ethanolic extract of *Boerhaavia diffusa* (BDE), a green leafy vegetable against mitochondrial dysfunction in angiotensin II (Ang II) induced hypertrophy in H9c2 cardiomyoblasts was evaluated. H9c2 cells challenged with Ang II exhibited pathological hypertrophic responses and mitochondrial dysfunction which was evident from increment in cell volume (49.09±1.13%), protein content (55.17±1.19%), LDH leakage (58.74±1.87%), increased intracellular ROS production (26.25±0.91%), mitochondrial superoxide generation (65.06±2.27%), alteration in mitochondrial transmembrane potential (ΔΨm), opening of mitochondrial permeability transition pore (mPTP) and mitochondrial swelling. In addition, activities of mitochondrial respiratory chain complexes (I-IV), aconitase, NADPH oxidase, thioredoxin reductase, oxygen consumption rate and calcium homeostasis were evaluated. Treatment with BDE significantly prevented the generation of intracellular ROS and mitochondrial superoxide radicals and protected the mitochondria by preventing dissipation of ΔΨm, opening of mPTP, mitochondrial swelling and enhanced the activities of respiratory chain complexes and oxygen consumption rate in H9c2 cells. Activities of aconitase and thioredoxin reductase which was lowered (33.77±0.68% & 45.81±0.71% respectively) due to hypertrophy, were increased in BDE treated cells (*P*≤0.05). Moreover, BDE also reduced the intracellular calcium overload in Ang II treated cells. Overall results revealed the protective effects of *B. diffusa* against mitochondrial dysfunction in hypertrophy in H9c2 cells and the present findings may shed new light on the therapeutic potential of *B. diffusa* in addition to its nutraceutical potentials.

## Introduction

Heart diseases are one of the leading causes of death worldwide [1]. Hypertension accounts a major risk for the development of cardiac diseases through induction of left ventricular hypertrophy and this ultimately leads to congestive heart failure and death [2]. Cardiac hypertrophy is the enlargement of heart with increase in the volume of cardiac cells and prolonged hypertrophic status has been associated with decompensation of heart function, development of heart failure and sudden death in humans [3]. Oxidative stress induced by various free radicals plays a vital role in the development of cardiac hypertrophy [4]. Mitochondria represent a substantial proportion (∼30%) of the heart cell’s mass and mitochondrial dysfunction is usually associated with pathological hypertrophy [5]. Dysfunctional mitochondria act as one of the most significant sources of reactive oxygen species (ROS) production in the heart [6]. Angiotensin II is a major component of rennin-angiotensin system that plays a key role in the development of left ventricular hypertrophy [7]. It has been shown that angiotensin II stimulate mitochondrial dysfunction in cardiac cells and subsequently produce excessive amounts of ROS such as superoxide, hydrogen peroxide, and peroxynitrite. This overproduction of mitochondrial ROS has been implicated in heart failure [8]. Since mitochondrial dysfunction plays a critical role in the development of cardiac hypertrophy and heart failure, the mitochondria is emerging as one of the important druggable targets in the management of cardiac hypertrophy and other associated complications.

Natural products are becoming popular throughout the world and widely accepted as an adjunct to conventional therapy [9]. Various epidemiological, experimental and clinical studies have revealed that natural products in the form of functional foods or nutracuticals play an important role in the prevention and management of cardiac diseases in prophylactic way [10], [11]. High consumption of plant-based foods is associated with a significantly lower risk of coronary artery disease most likely due to the abundance and variety of bioactive compounds present in it [12], [13]. Besides antioxidant activity, natural products have other biological properties like lipid lowering, antihyperglycemic, antihypertensive etc. that lead to reduce the risk of cardiovascular disorders.

*Boerhaavia diffusa* L. from the family *Nyctaginaceae* is widely used as green leafy vegetable and an important indigenous medicinal plant with lots of biological properties. The plant is reported to possess cardiotonic and antihypertensive potential [14], [15]. Pharmacological studies have demonstrated that *B. diffusa* possess antioxidant [16], antidiabetic [17], immunomodulatory [18], anticonvulsant, hepatoprotective, antibacterial, antiproliferative and antiestrogenic activities [19], [20]. Our previous studies showed the antihypertrophic potential of *B. diffusa* against angiotensin II induced hypertrophy in H9c2 cells by down regulating oxidative stress along with its potent antioxidant capacity [21].

The present study aims to evaluate the mitochondrial dysfunction in angiotensin II induced hypertrophy in H9c2 cells and the protective effects of *B. diffusa* against mitochondrial damage in cardiac hypertrophy.

## Materials and Methods

*B. diffusa* were collected from local areas of Thiruvananthapuram, India, identified and authenticated by Dr. H. Biju, Taxonomist, Jawaharlal Nehru Tropical Botanic Garden Research Institute (JNTBGRI), Palode, Thiruvananthapuram, Kerala. No specific permissions were required for the collection of this plant. Plant material is plenty available, widely distributed and is not an endangered or protected species and the GPS coordinates of location of plant collection is 8° 27' 36" North, 76° 59' 41" East. A voucher specimen was kept in our herbarium for future reference (No. 01/05/2010 APNP/CSIR-NIIST). Extraction of the whole plant material was done with ethanol as per our previous reports [21] and the yield of the *B. diffusa* extract (BDE) was found to be 12.64% (w/w). The same lot of the extract was used to conduct all the experiments.

### Cell culture and treatment

The H9c2 embryonic rat heart-derived cell line was obtained from the American Type Culture Collection (ATCC) and were cultured in Dulbecco’s modified eagle medium (HiMedia, India) containing 4.5 g/L glucose, 1.5 g/L sodium bicarbonate and 110 mg/L sodium pyruvate, supplemented with 10% fetal bovine serum (Gibco, New Zealand) and penicillin (100 units/ml) and streptomycin (100 µg/ml) in a humidified incubator with 95% air and 5% CO_2_ at 37°C. The culture medium was changed every 2 days. Then the cells were passaged and seeded at the density of 3×10^5^ cells/cm^2^ growth area of T75 (75 cm^2^) tissue culture flask or 1.2×10^6^ cells per 100 mm dish or 0.64 ×10^4^ cells per 6.4 mm well of 96 well plates. These cells were cultured for 3 days and then underwent treatments.

H9c2 cells were treated with BDE for 6 hrs prior to angiotensin II (Ang II) treatment. Ang II (100 nM) (Sigma-Aldrich, St. Louis, MO, USA) was prepared in double distilled water and diluted with culture media to induce hypertrophy and cultured for an additional 48 hrs [21]. The experimental group consist of (a) Control cells (b) BDE (75 µg/ml) alone treated cells, (c) Ang II (100 nm) alone treated cells, (d) BDE (75 µg/ml) + Ang II (100 nm) treated cells. Dose of the Ang II and BDE was selected based on our previous studies [21].

Induction of hypertrophy was confirmed by determining cell volume, protein content and LDH leakage [21].

### Detection of intracellular reactive oxygen species (ROS) and mitochondrial superoxide production

Intracellular ROS levels were measured using flow cytometry with fluorescent 2’, 7’ dichlorodihydrofluorescein diacetate (DCFH-DA) as probe [22]. DCFH-DA is cleaved intracellularly by non-specific esterase and turn to high fluorescent upon oxidation by ROS, which were analyzed with FACS Aria II (BD Bioscience, San Jose, USA).

Mitochondrial superoxide productions in the live cells were evaluated with fluorescent dye, mitoSOX. Briefly after respective treatments, cells were loaded with mitoSOX (5 µM) in the medium and incubated for 20 minutes. For bioimaging (BD Pathway™ Bioimager System, BD Biosciences), the dye was excited at 514 nm as described earlier [23].

### Activities of aconitase, thioredoxin reductase, xanthine oxidase and NADPH oxidase

Activity of aconitase, thioredoxin reductase and xanthine oxidase was assayed in control and treated cells using respective kits from Cayman chemicals (USA) as per manufacturer’s instructions. Activity of NADPH oxidase was done as per the method of Qin et al., (2006) [24].

### Determination of mitochondrial transmembrane potential (ΔΨm), integrity of mitochondrial permeability transition pore (mPTP) and mitochondrial swelling

Change in ΔΨm was detected using a mitochondria staining kit (Sigma-Aldrich, St. Louis, MO, USA) that uses JC-1, a cationic fluorescent dye. Briefly, the cells were seeded in 96-well black plates at a density of 5×10^3^ cells per well. After 48 hours of treatment, the cells were incubated with JC-1 stain and incubated for 20 minutes. For imaging of JC-1 monomers, the live cell bioimager (BD Pathway™ Bioimager System, BD Biosciences) was set at 490 nm excitation and 530 nm emission wavelengths, and for J- aggregates, the wavelengths were set at 525 nm excitation and 590 nm emission [25]. Valinomycin was used as positive control.

To examine the mPTP opening, the cells were loaded with calcein-AM (0.25 µM) in the presence of 8 mM cobalt chloride for 30 minutes to quench cytosolic and nuclear calcein fluorescence [25]. The calcein fluorescence is then compartmentalized within mitochondria until PTP opening permits the distribution of cobalt inside mitochondria, which results in the quenching of calcein fluorescence in the mitochondrial matrix. The PTP opening thus leads to the decompartmentalization of calcein fluorescence. Images of cells were taken at 488 nm excitation and 525 nm emissions (BD Pathway™ Bioimager System, BD Biosciences).

For the determination of mitochondrial swelling, mitochondria were isolated using a mitochondrial isolation kit from Sigma-Aldrich, (St. Louis, MO, USA). Mitochondrial swelling was determined as per previously described method [26]. In brief, mitochondria (1 mg/ml) were incubated in a total volume of 1.8 ml of respiratory buffer (125 mM sucrose, 50 mM KCl, 5 mM HEPES, 2 mM KH_2_PO_4_, 1 mM MgCl_2_ at pH 7.2) in the presence of 6 mM succinate at 25°C. Rotenone (2 µM) was added to the buffer just before the experiment. CaCl_2_ (100 µM) was used as swelling agent. The change in absorbance was measured at 540 nm and the decrease in absorbance indicates the increase in mitochondrial swelling.

### Determination of the activity of mitochondrial respiratory complexes and oxygen consumption assay

After respective treatments, mitochondria were isolated using a mitochondrial isolation kit (Sigma-Aldrich, St. Louis, MO, USA) and suspended in 50 mM/L phosphate buffer (pH 7.0). Then it was frozen and thawed 3–5 times to release the enzymes (except complex IV, which was extracted with 0.5% Tween 80 in phosphate buffer, v/v). The effect of BDE on complex I-mediated electron transfer (NADH dehydrogenase) was studied using NADH as the substrate and menadione as electron acceptor. The reaction mixture containing 200 µM menadione and 150 µM NADH was prepared in phosphate buffer (0.1 M, pH 8.0). To this mitochondria (100 µg) was added, mixed immediately and observed quickly for change in the absorbance at 340 nm for 8 minutes (UV-2450 PC; Shimadzu, Kyoto, Japan) [27]. Rotenone (10 ìM) was used to inhibit the complex I.

Complex II mediated activity (succinate dehydrogenase) was measured spectrophotometrically at 600 nm using dichlorophenolindophenol (DCPIP) as an artificial electron acceptor and succinate as substrate. The extent of decrease of absorbance (ΔOD) was considered as the measure of the electron transfer activity of complex II [27]. The reaction mixture was prepared in 0.1 M phosphate buffer (pH 7.4) containing 10 mM EDTA, 50 µM DCPIP, 20 mM succinate and mitochondria (50 µg). The change in absorbance was observed immediately for 8 minutes at 30°C. Malonate (25 µM) was used to inhibit the complex II.

Complex III (Ubiquinol-cytochrome c reductase) activity was determined as per the method described previously [28]. In brief mitochondrial protein (50 µg) was mixed with 100 µM/L EDTA, 2 mg BSA, 3 mmol/L sodium azide, 60 µM/L ferricytochrome C, decylubiquinol (1.3 mM) and phosphate buffer (50 mM, pH 8) in a final volume of 1 ml. The reaction was started by the addition of decylubiquinol and monitored for 2 min at 550 nm and again after the addition of 1 µmol/l of antimycin A. The activity was calculated from the linear part of absorption–time curve, which was not less than 30 seconds. Activity of complex III was expressed as μmoles of ferricytochrome C reduced/min/mg protein. Antimycin A (10 µM) was used as standard inhibitor of complex III.

Activity of complex IV (cytochrome C oxidase) was determined as per previous method [28]. Briefly 1 ml of ferrocytochrome C solution was mixed with approximately 10 µg of mitochondrial protein (extracted in 0.5% Tween 80 in 30 mmol/L phosphate buffer, pH 7.4) and phosphate buffer in a net volume of 1.3 ml. The reaction was started by the addition of enzyme source and was monitored at 550 nm with an interval of 15 seconds for 4 min. The difference in absorbance was calculated from the linear part of the absorption-time curve. KCN (5 µM) was used as inhibitor of complex IV. Complex (IV) activity was expressed as micromoles of ferrocytochrome C oxidized/min/mg protein using the extinction coefficient 21 mM^−1^ cm^−1^.

Oxygen consumption rate in control and treated cells were assayed using Cayman’s cell based oxygen consumption rate assay kit using antimycin A as standard inhibitor (Cayman Chemicals, Ann Arbor, USA)

### Intracellular calcium ([Ca^2+^]i) overload and the activity of calcium ATPase

[Ca^2+^]i overload was detected by staining the cells after respective treatments with Fura-2AM for 20 min at 37°C and the images were visualized using BD Pathway™ Bioimager System; BD Biosciences [29].

Activity of calcium ATPase was evaluated as per previous method [30]. In this assay, 0.1 ml of cell lysate was added to the reaction mixture composed of 0.4 M Tris HCl, 15 mM NaN_3_, 0.2 mM EDTA, 120 mM CaCl_2_, 20 mM MgCl_2_ to all the tubes. Then 0.2 ml of ATP (3 mM as substrate) was added to the test tubes. All the tubes were incubated for 30 min in a water bath at 37°C and the enzyme activity was stopped by adding 2 ml of 10% trichloroacetic acid (TCA). All the tubes were then centrifuged at 2,500 rpm for 10 minutes to collect supernatant. The protein-free supernatant was then analyzed for inorganic phosphate. For that 3 ml of the supernatant was treated with 1 ml of ammonium molybdate and 0.4 ml of 1-amino 2-naphthol 4-sulphonic acid (ANSA) and then absorbance was read at 680 nm after 20 min.

### Statistical analysis

Results were expressed as means and standard deviations (SD) of the control and treated cells from three independent experiments in duplicates (n = 6). Data were subjected to one-way ANOVA and the significance of differences between means was calculated by Duncan’s multiple range test using SPSS for Windows, standard version 11.5.1 (SPSS, Inc.), and significance was accepted at *P*≤0·05.

## Results

### Cell volume, protein content and LDH leakage in control and hypertrophied cells

Induction of hypertrophy by Ang II in H9c2 cells was confirmed by measuring cell volume, protein content and LDH leakage (Table 1). Ang II treated cells showed increased cell volume (49.09±1.13% increase), protein content (55.17±1.19% increase) and LDH leakage (58.74±1.87% increase) than normal cells confirming the induction of hypertrophy. Treatment with BDE significantly reduced the genesis of hypertrophy by Ang II in H9c2 cells. There was no significant increase in cell volume and protein content in H9c2 cells pretreated with BDE alone when compared to normal cells.

**Table 1:** Change in cell volume, protein content and LDH leakage in different groups.

Parameters	Control	BDE alone	Ang II	Ang II + BDE
Cell volume (μm^3^×10^3^)	2.05±0.12	2.12±0.18	3.21±0.15^*^	2.65±0.14^**^
Protein content (mgX10^6^ Cells)	0.29±0.017	0.301±0.025	0.45±0.02^*^	0.37±0.017^**^
LDH leakage (μU/ml)	5.89±0.72	5.95±0.81	10.05±1.02^*^	6.52±0.54^**^

Values expressed as mean ± SD (n = 6). * indicates significant difference from control cells and ** indicates significant difference from Ang II treated hypertrophied cells and the significance accepted at *P*≤0·05.

### Effect of BDE on intracellular ROS and mitochondrial superoxide production

**Figure 1:**
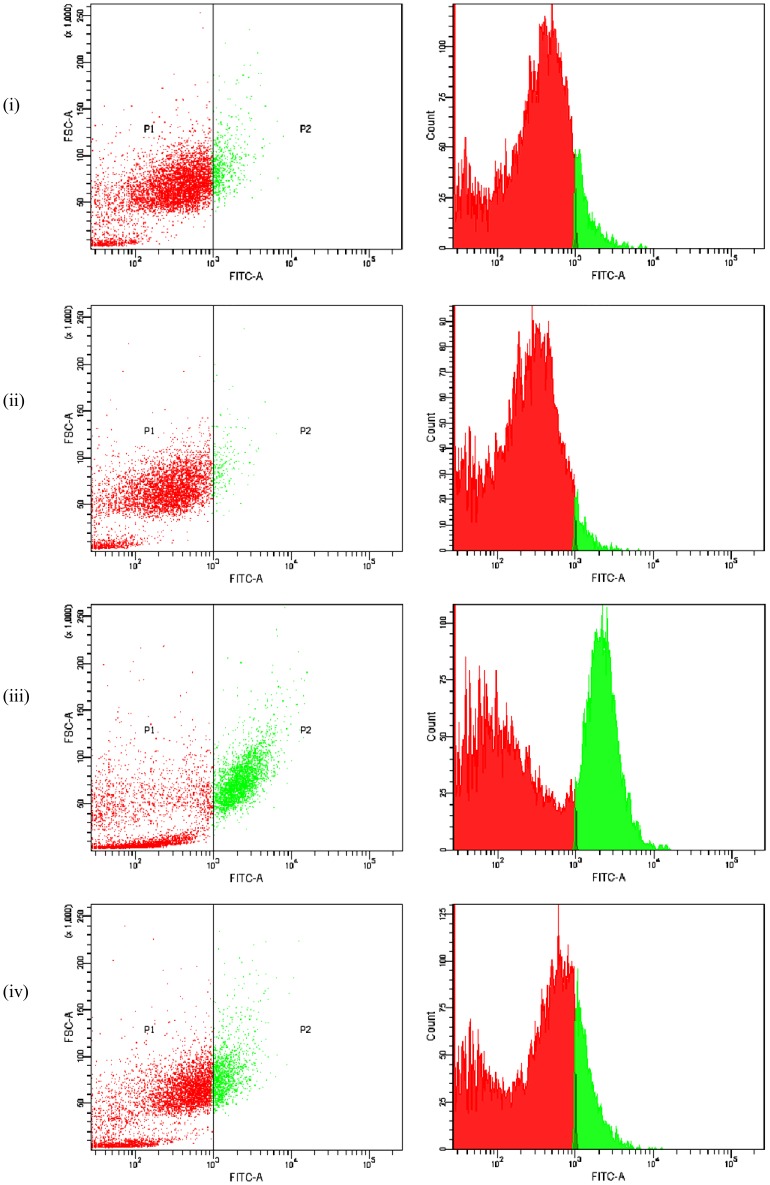
Flow cytometric analysis of intracellular ROS generation in different groups. Analysis of intracellular ROS using fluorescent probe, 2’,7’-dichlorfluorescein-diacetate (DCFH-DA) reveals significant increase in ROS generation by Ang II but BDE treatment curtails the same on Ang II application. (i) Control cells (ii) BDE alone treated cells (75µg/ml) (iii) Ang II (100 nm) treated cells (iv) BDE+Ang II treated cells. Population P2 represents the ROS. Results expressed as mean ± SD; n = 6 and the significance accepted at (*P*≤0.05).

Flow cytometry analysis of ROS showed that Ang II significantly (*P*≤0.05) elevated the intracellular ROS level (26.25±0.91%) in H9c2 cells than that of control (Fig. 1). Ang II induced ROS generation was significantly reduced (*P*≤0·05) by the treatment with BDE when compared to Ang II alone treated cells.

**Figure 2:**
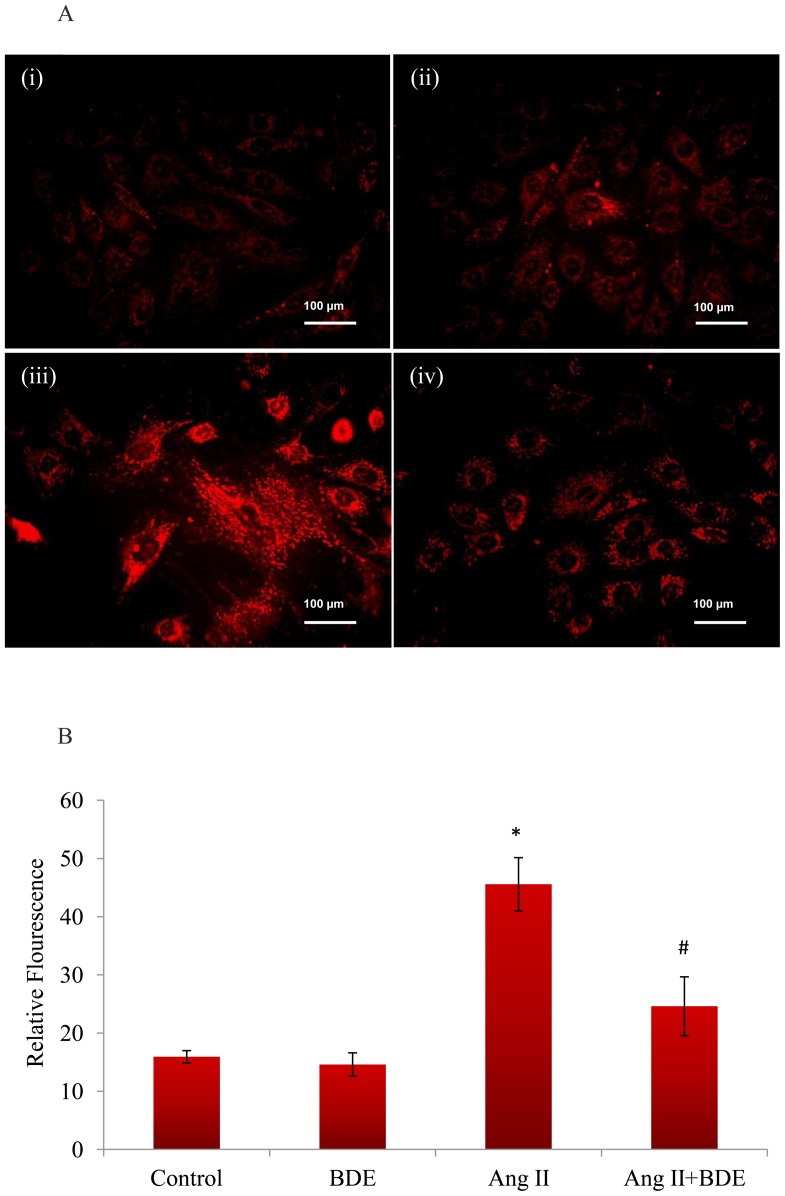
Mitochondrial superoxide radical generation in different groups. The representative images of the cells after treatment with Ang II and BDE stained with mitoSOX. Ang II (100 nm) caused surplus superoxide generation in H9c2 cells whereas BDE treatment (75µg/ml) reduced Ang II induced superoxide production in the cells (**A**). (i) Control cells, (ii) BDE alone treated cells, (iii) Ang II treated cells and (iv) BDE+Ang II treated cells. Intensity of fluorescence emitted by mitoSOX in control and treated cells (B). Results expressed as mean ± SD; n = 6. * indicates significant difference from control cells (*P*≤0.05) and # indicates significant difference between BDE+Ang II and Ang II alone treated cells (*P*≤0.05).

In addition, there was an increased generation of mitochondrial superoxide radicals (65.06±2.27%) in hypertrophied cells compared to control cells while BDE treatment significantly reduced the generation of superoxide radicals to 46.03±1.78% (Fig. 2A & 2B) when compared with hypertrophied cells.

### Activities of aconitase, thioredoxin reductase, xanthine oxidase and NADPH oxidase

Activities of aconitase and thioredoxin reductase were significantly reduced in Ang II induced hypertrophied cells (33.77±0.68% & 45.81±0.71% respectively) whereas activities of xanthine oxidase and NADPH oxidase were significantly elevated (84.17±0.87 & 137.78±0.93% respectively) when compared with control cells. BDE treatment reversed these changes significantly (*P*≤0.05) and brought back the activity near to normal (Table 2).

**Table 2:** Activities of aconitase, thioredoxin reductase, xanthine oxidase and NADPH oxidase.

Enzymes	Control	BDE alone	Ang II	Ang II+BDE
Aconitase (nm/min/ml)	6.81±0.42	6.77±0.51	4.19±0.35^*^	5.92±0.29^#^
Thioredoxin reductase (μM/min/ml)	0.029±0.002	0.028±0.001	0.009±0.004^*^	0.022±0.003^#^
Xanthine oxidase (relative fluorescence)	990.96±79.57	987.02±72.78	1825.09±121.14^*^	1293.76±57.89^#^
NADPH oxidase (nM/mg protein)	1.35±0.05	1.41±0.09	3.21±0.27^*^	2.29±0.19^#^

Values expressed as mean ± SD (n = 6). * indicates significant difference from control cells and # indicates significant difference from Ang II treated hypertrophied cells and the significance accepted at *P*≤0·05.

### Effects of BDE on ΔΨm and mPTP and mitochondrial swelling

**Figure 3:**
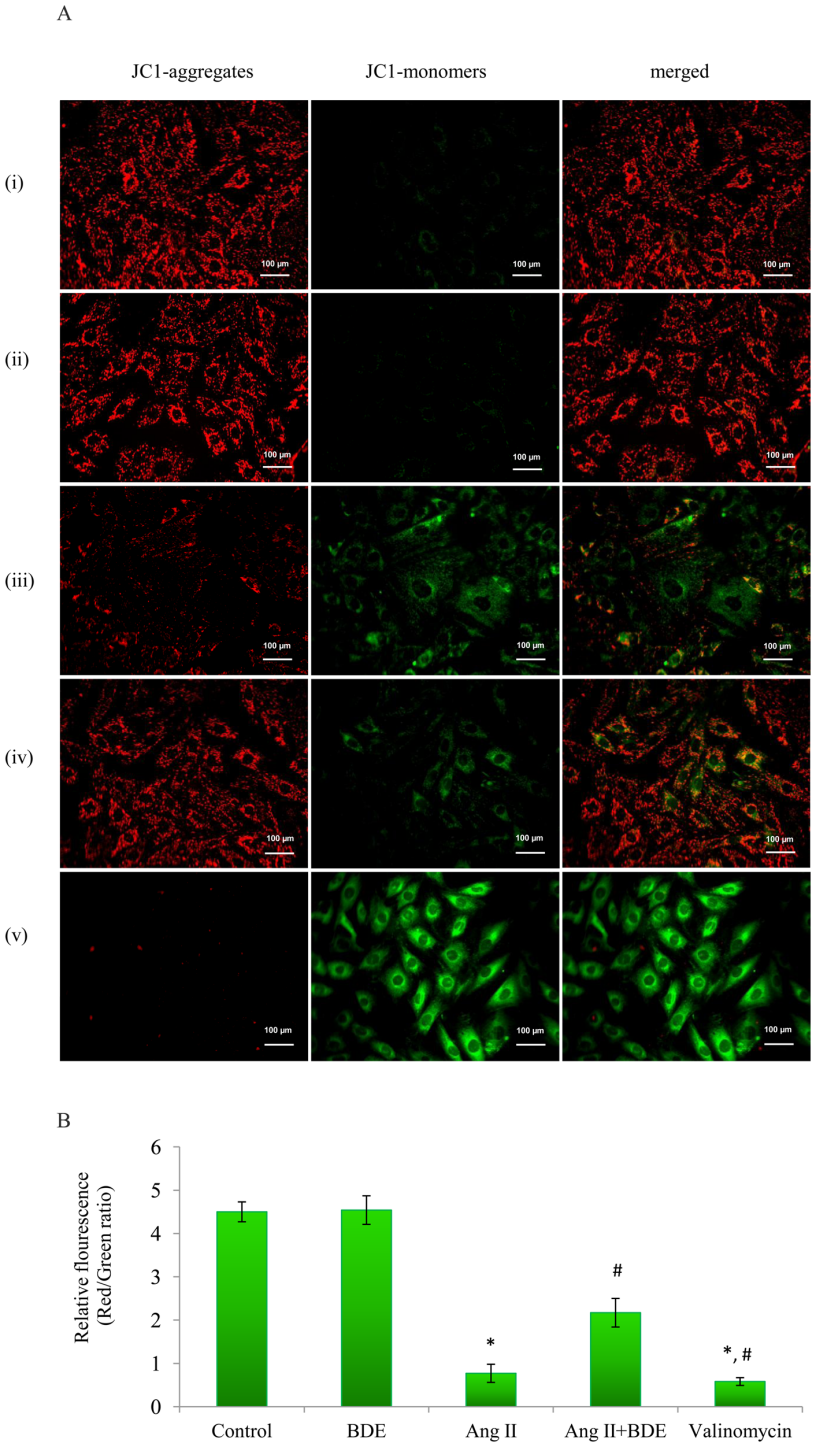
Change in mitochondrial transmembrane potential (ΔΨm) in different groups. The representative images show JC-1 aggregates, JC-1 monomers and merged images of both (**A**). JC-1 aggregates are more in control cells revealing intact mitochondria and the formation of JC-1 monomers in Ang II treated cells shows dissipation of ΔΨm. Decrease in JC-1 monomers and increase in JC-1 aggregates in BDE treated hypertrophied cells shows the capability of extract to protect mitochondria. (i) Control cells, (ii) BDE (75µg/ml) alone treated cells, (iii) Ang II (100 nm) treated cells and (iv) BDE+Ang II treated cells (v) valinomycin treated cells. Ratio of JC-1 aggregates to JC-1 monomers (B). The graphical representation of the ratio of JC-1 aggregates to JC-1 monomers (ratio of 590:530 nm emission intensity) reveal ΔΨm dissipation with Ang II and restoration by BDE. Results expressed as mean ± SD; n = 6. * indicates significant difference from control cells (*P*≤0.05) and # indicates significant difference between BDE+Ang II and Ang II alone treated cells (*P*≤ 0.05).

Fig. 3A & 3B show the mitochondrial transmembrane potential of control and treated cells. The JC-1 dye concentrates in mitochondrial matrix and form red fluorescent aggregates in normal cells due to the existence of electrochemical potential gradient. Alteration of ΔΨm prevents the accumulation of JC-1 in the mitochondria and gets dispersed throughout the cells, leading to a shift from red (JC-1 aggregates) to green fluorescence (JC-1 monomers). Hypertrophied cells exhibited depolarized ΔΨm which was evident from significantly higher amount of JC-1 monomers (green fluorescence). On the other hand, BDE treatment prevented the alteration of ΔΨm which was clearly evident from the increased level of JC-1 aggregates (red fluorescence).

**Figure 4:**
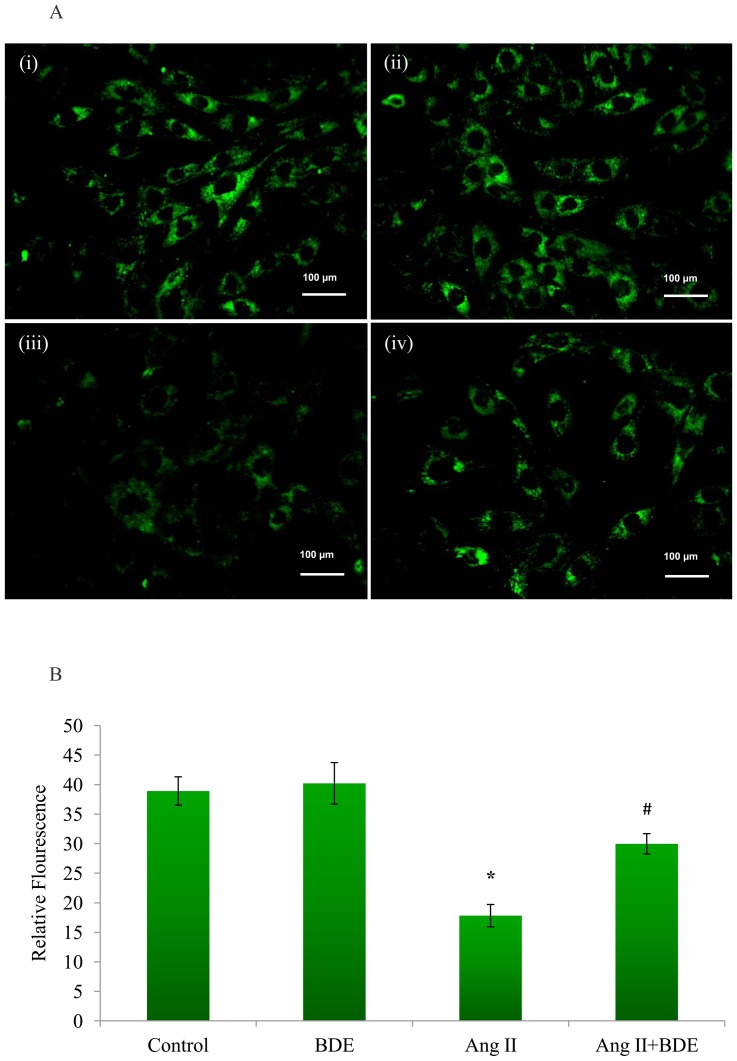
Alteration in the integrity of mitochondrial permeability transition pore (mPTP). Fluorescent images of the cells show the significant change in integrity of mPTP with Ang II and the protection by BDE (**A**). Reduced fluorescence in hypertrophied cells indicates opening of mitochondrial permeability transition pore and BDE treatment reversed these changes induced by Ang II. (i) Control cells, (ii) BDE (75µg/ml) alone treated cells, (iii) Ang II (100 nm) treated cells and (iv) Ang II+ BDE treated cells. Intensity of fluorescence emitted by calcien-AM in control and treated cells (B). Results expressed as mean ± SD; n = 6. * indicates significant difference from control cells (*P*≤0.05) and # indicates significant difference between BDE+Ang II and Ang II alone treated cells (*P*≤0.05).

The opening of mPTP was examined using calcein-AM staining combined with CoCl_2_. Calcein-AM freely passes through cellular membranes, and the esterases in the cells cleave the acetomethoxy group to yield the fluorescent calcein. Co-loading of cells with CoCl_2_ quenches the fluorescence in the cell, except in mitochondria, since CoCl_2_ cannot cross mitochondrial membrane. Therefore, during the opening of mPTP, mitochondrial calcein is also quenched by CoCl_2_, resulting in reduced fluorescence [31], [32]. Integrity of mPTP was altered significantly in Ang II treated hypertrophied cells compared to control cells which was evident from reduced calcein fluorescence (Fig. 4A & 4B). Presence of BDE protected the integrity mPTP in Ang II treated H9c2 cells.

**Figure 5:**
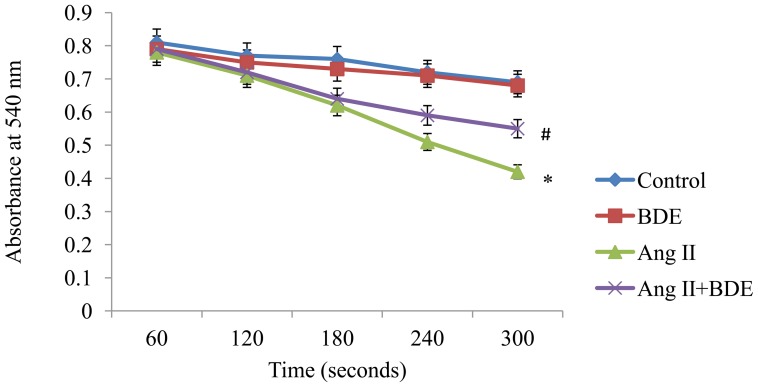
Mitochondrial swelling in different groups. The graphical representation shows the increase of mitochondrial swelling in Ang II induced hypertrophied cells and its prevention by BDE pre-treatment. Results expressed as mean ± SD; n = 6. * indicates significant difference from control cells (*P*≤0.05) and # indicates significant difference between BDE+Ang II and Ang II alone treated cells (*P*≤0.05).

Investigation on mitochondrial swelling is one of the methods for the assessment of mitochondrial membrane integrity. H9c2 cells exposed to Ang II showed increased mitochondrial swelling than control cells (Fig. 5) whereas BDE treatment reduced the swelling of mitochondria significantly when compared with hypertrophied cells (*P*≤0.05).

### Activities of mitochondrial respiratory complexes

Table 3 shows the activities of mitochondrial respiratory complexes in control and treated cells. The activities of respiratory chain complexes such as complexes I, II, III and IV were significantly decreased in Ang II treated rats (*P*≤0.05) compared to control cells. There were 47.93±0.16, 28.28±0.13, 48.94±0.19 and 30.71±0.85% reduction in the activities of complexes I, II, III and IV respectively in hypertrophied cells whereas BDE treatment prevented the reduction on the activities of respiratory chain complexes in Ang II exposed H9c2 cells (p≤0.05). There were 68.87±0.41, 21.62±0.85, 59.74±0.78 and 25.20±0.69% increase in the activities of respiratory chain complexes I, II, III and IV respectively in BDE treated cells when compared to hypertrophied cells. Standard compounds like rotenone, inhibited complex I activity by 62.06±0.64%, malonate inhibited complex II activity by 66.13±0.77%, antimycin A inhibited complex III activity by 72.01±0.91% and KCN inhibited complex IV activity by 82.34±0.97% when compared to control cells.

**Table 3:** Activities of mitochondrial respiratory complexes in control and treated cells.

	Complex I (ΔOD 340 nm)	Complex II (ΔOD 600 nm)	Complex III (μM of ferricytochrome C reduced/min/mg protein)	Complex IV (μM of ferrocytochrome C oxidized/min/mg protein)
Control	0.291±0.005	0.251±0.009	7.54±1.05	5.21±0.68
BDE alone	0.306±0.012	0.245±0.011	7.39±1.25	5.30±0.95
Ang II	0.151±0.015^*^	0.183±0.016^*^	3.75±1.31^*^	3.61±1.04^*^
Ang II+BDE	0.255±0.007^#^	0.219±0.008^#^	6.15±1.52^#^	4.52±0.61^#^
Rotenone	0.111±0.008^*^			
Malonate		0.085±0.021^*^		
Antimycin A			2.11±0.34^*^	
KCN				0.92±0.12^*^

Values expressed as mean ± SD (n = 6). * indicates significant difference from control cells and # indicates significant difference from Ang II treated hypertrophied cells and the significance accepted at *P*≤0·05.

### Oxygen consumption rate in control and treated cells

**Figure 6:**
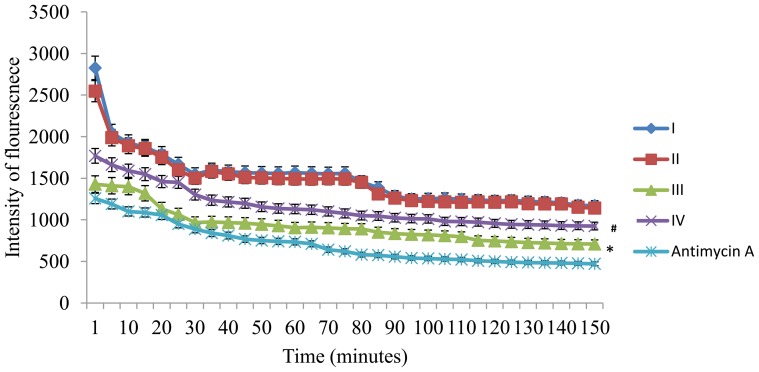
Oxygen consumption rate in different groups. Reduction in fluorescence indicates lower oxygen consumption rate in the cells. Results expressed as mean ± SD; n = 6. * indicates significant difference from group I and # indicates significant difference from group III (*P*≤0.05). Group I- Control cells; Group II-BDE (75µg/ml) alone treated cells; Group III-Ang II (100 nm) treated cells; Group IV-Ang II+BDE treated cells.

Oxygen consumption rate in living cells were analyzed by using a phosphorescent probe, mitoXpress and the reduction in fluorescent/phosphorescent signal over time indicates lower oxygen consumption rate in the cells. Hypertrophied cells showed reduced oxygen consumption rate when compared to control cells and treatment with BDE reversed these changes near to normal (*P*≤0.05) indicates BDE protects against mitochondrial dysfunction in hypertrophy (Fig. 6).

### [Ca^2+^]i overload and the activity of calcium ATPase

**Figure 7:**
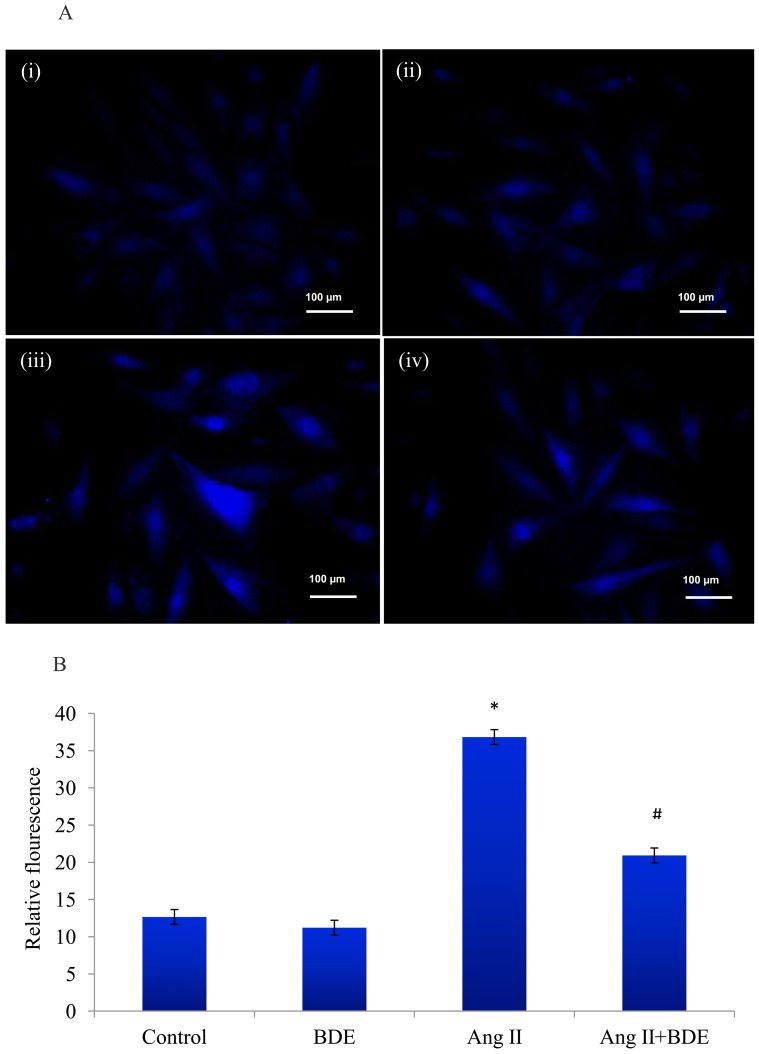
Effect of BDE on intracellular calcium overload in different groups. The representative images shows higher fluorescence in the cells treated with Ang II indicates calcium overload whereas reduced fluorescence in BDE treated cells indicates the reduction in calcium overload (**A**). (i) Control cells, (ii) BDE (75µg/ml) alone treated cells, (iii) Ang II (100 nm) treated cells and (iv) BDE+Ang II treated cells. Intensity of fura-2AM fluorescence in control and treated cells (B). High intensity of fura-2AM in Ang II treated cells indicates calcium overload in hypertrophy whereas reduced fluorescence in BDE treatment shows the inhibition of calcium overload. Results expressed as mean ± SD; n = 6. * indicates significant difference from control cells (*P*≤0.05) and # indicates significant difference between Ang II+ BDE and Ang II alone treated cells (*P*≤0.05).

**Figure 8:**
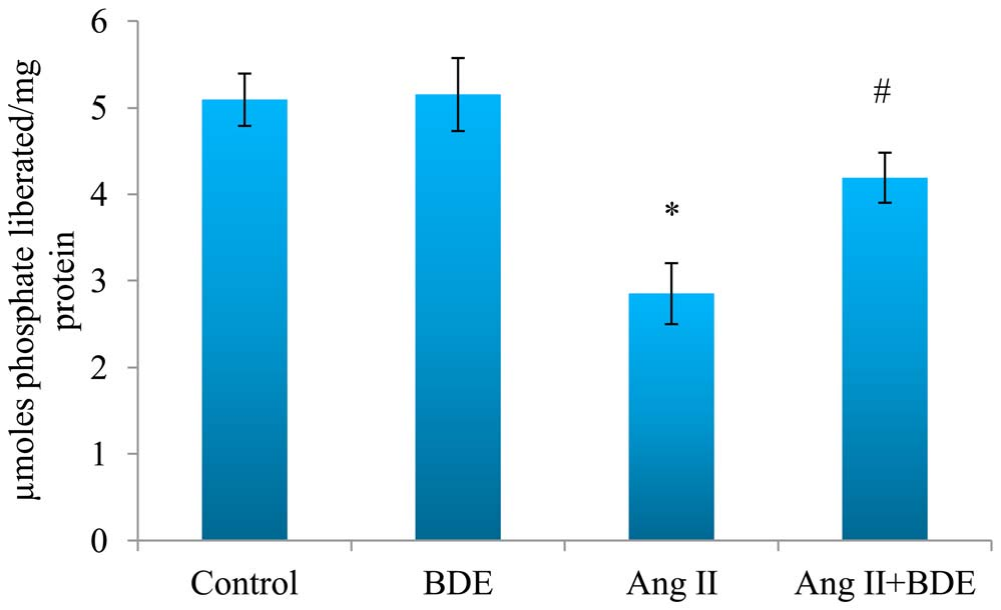
Activity of Ca^2+^ATPase in different groups. The graphical representation shows the reduction of Ca^2+^ATPase activity in hypertrophied cells whereas BDE treatment protected the enzyme from deleterious effect of Ang II. Results expressed as mean ± SD; n = 6. * indicates significant difference from control cells (*P*≤0.05) and # indicates significant difference between BDE+Ang II and Ang II alone treated cells (*P*≤0.05).

Ang II induced [Ca^2+^]i overload in H9c2 cells which was evident from increased Fura-2AM fluorescence (Fig. 7A & 7B) whereas activity of calcium ATPase (Fig. 8) was significantly reduced (p≤0.05). Treatment with BDE reduced [Ca^2+^]i overload and brought back the activity of calcium ATPase (p≤0.05) near to normal level. This suggests that BDE positively modulates the calcium homeostasis in hypertrophied cardiac myoblasts.

## Discussion

Alteration in mitochondrial function plays a key role in the pathogenesis of cardiac hypertrophy that may ultimately leads to heart failure [6]. The heart has continuous demands for high energy and the adequate supply of ATP is critical for electrical and mechanical functions of heart [33]. Over 90% of energy consumption of the heart is from mitochondria and it plays key role in many cellular functions including energy production, calcium homeostasis and cell signalling [34]. Recent reports reveal that crisis in energy production due to impaired mitochondrial function would result in cardiometabolic diseases [35]. Recently, the significance of metabolic remodelling process in the hypertrophic growth response of the heart has been identified [36]. All these information categorically declare the profound importance of mitochondria in cardiac hypertrophy and other heart disorders. Mitochondria, the major site of ROS generation as a by product of oxidative phosphorylation and ROS plays a critical role in the development of Ang II induced cardiac hypertrophy [7]. Significant changes in mitochondrial function as well as mitochondrial energetics have been described in various forms of cardiac hypertrophy [37]. Swollen cardiac mitochondria with disrupted cristae and substantial mitochondrial DNA depletion along with reduction in the activities of respiratory chain complexes were also observed in hypertrophic cardiomyopathy [38]. The possible potential mechanisms of mitochondrial dysfunction in pathological hypertrophy include ROS, cardiolipin loss or peroxidation, mitochondrial uncoupling, impaired mitochondrial biogenesis, reduced transcriptional signalling of regulators of mitochondria etc. [37].

The present study demonstrates for the first time that ethanolic extract of *B. diffusa* (BDE) attenuates hypertrophy induced mitochondrial dysfunction in heart-derived H9c2 cells. Our previous studies have revealed that BDE protects H9c2 cardiomyoblasts against Ang II induced hypertrophy via its potent antioxidant activity [21]. Elevated levels of intracellular ROS (Fig. 1) along with surplus generation of mitochondrial superoxide radicals in hypertrophied cells (Fig. 2A & 2B) indicate the development of oxidative stress during hypertrophy. Increased superoxide radical generation affect the normal functioning of mitochondria and that to the progression of left ventricular hypertrophy [39]. Reduced generation of intracellular ROS and mitochondrial superoxide radicals in BDE treated cells shows the free radical scavenging potential of the extract (Fig. 1, 2A & 2B). NADPH oxidase and xanthine oxidase are two important enzymes that play significant role in cardiovascular pathology and these are the major enzymatic source of ROS in cardiovascular system [40], [41]. Increase in the activities of these enzymes leads to increased production of superoxide radicals that ultimately lead to cardiac dysfunction [42]. Previous reports also suggest that NADPH dependant superoxide radical generation is associated with the development of cardiac hypertrophy [24] and the increased production of mitochondrial ROS by Ang II is mediated through NADPH oxidase [8]. It is interesting to note that treatment with BDE significantly prevented the alteration of these enzymes in the cells exposed with Ang II. Reduced activities of aconitase and thioredoxin reductase in hypertrophied cells again indicate mitochondrial dysfunction via excessive production of ROS. Reduced activity of mitochondrial aconitase is an indicator of mitochondrial superoxide production [43] and there is an inverse relation between superoxide production and activity of aconitase in cardiac hypertrophy [44]. Reports suggest that thioredoxin reductase can attenuate cardiac hypertrophy not only by scavenging ROS but also involved in several steps of redox regulation of cell [45]. Here also BDE treatment restored the activities of aconitase and thioredoxin reductase in hypertrophied cells.

ΔΨm is essential for normal mitochondrial function and dissipation of ΔΨm indicates mitochondrial dysfunction [25]. Mitochondrial permeability transition is involved in the control of mitochondrial calcium homeostasis and apoptosis [46] and swelling of mitochondria is known to correlate with mitochondrial dysfunction and damage [37]. The present study reveals significant changes in ΔΨm (depolarization) (Fig. 3A & 3B), integrity of mPTP (Fig. 4A & 4B) and mitochondrial swelling (Fig. 5) in hypertrophied cell. Depolarization of ΔΨm by Ang II was dependent on increased NADPH oxidase activity and ROS [8]. Alteration in ΔΨm may lead to the uncoupling of respiratory chain, and this accompanies mPTP opening [46] and the activation of mPTP opening disrupts the permeability barrier of the inner mitochondrial membrane, causing uncoupling of oxidative phosphorylation, osmotic swelling, and rupture of the outer membrane and ultimately cell death [34], [47]. One of the main events that are thought to trigger mitochondrial dysfunction is mPTP, with subsequent opening of the mitochondrial pore and mitochondrial swelling [48]. This is a clear cut indication of the role of mitochondria in angiotensin II mediated hypertrophy in heart. BDE treatment was found to prevent the changes in ΔΨm, mPTP and mitochondrial swelling significantly in Ang II induced hypertrophied cells suggest that BDE can attenuate mitochondrial alterations in hypertrophied cells.

Excessive production of ROS impairs the activities of respiratory chain complexes which are very important in the biology of heart [49]. Generally, the impairment of complex I and III activities may increase the electron leakage from the electron transport chain, generating more superoxide radicals and perpetuating a cycle of oxygen radical induced damage to mitochondrial membrane constituents [49]. Activities of mitochondrial respiratory complexes were significantly reduced in hypertrophied cells suggesting the role of oxidative stress and reduced activities of respiratory complexes is reported to increase mitochondrial ROS production [8]. A reduction in complex I enzyme activity leads to accumulation of electrons in the initial part of the transport chain which facilitates direct transfer of electrons to molecular oxygen that results in the generation of superoxide radicals [50]. In addition, superoxide radicals can react with nitric oxide radical to form highly toxic peroxynitrite radical which in turn can cause serious mitochondrial dysfunction by damaging respiratory complexes [8]. BDE treatment protected the activities of these electron transport chain complexes from the deleterious effect of Ang II on myoblasts.

Oxygen consumption rate is an important indicator of normal cellular function and unhealthy cells with dysfunctional mitochondria show a lower oxygen consumption rate when compared to healthy cells. Since most of the oxygen consumption is via. mitochondria, oxygen consumption rate has been used as a parameter to study mitochondrial function [51]. In our study, reduced oxygen consumption rate in hypertrophied cells further supports the mitochondrial dysfunction and BDE treatment attenuated the reduction in oxygen consumption in H9c2 cells (Fig. 6). Ang II reduces oxygen consumption [52] and there were reports that pathological hypertrophy is associated with mitochondrial dysfunction and reduced oxygen consumption [37] and Ang II.

Mitochondria play an important role in cellular Ca^2+^ homeostasis [53]. [Ca^2+^]i overload, as a consequence of dysregulation of Ca^2+^ homeostasis, leads to cardiac dysfunction and heart failure [54]. In our study, ([Ca^2+^]i) overload and reduced activity of Ca^2+^ATPase in Ang II treated cells (Fig. 7A, 7B & 8) suggests the alteration of Ca^2+^ homeostasis in hypertrophy whereas treatment with BDE reversed these changes indicates that BDE can maintain calcium homeostasis in cardiac cells. Ca^2+^ ATPase is vital for regulating Ca^2+^ in the cell and [Ca^2+^]i overload is reported to trigger mPTP opening along with ROS that can ultimately leads to mitochondrial dysfunction [53]. In addition to this, [Ca^2+^]i overload can also enhance mitochondrial ROS production by increasing metabolic rate which in turn leads to respiratory chain electron leakage. Furthermore, Ca^2+^ can enhance the dislocation of cytochrome C from the mitochondrial inner membrane and this result in an effective block of the respiratory chain at complex III, which would enhance ROS generation [55].

Since mitochondrial oxidative damage plays significant role in cardiac dysfunction, protecting mitochondria from oxidative damage should be an effective therapeutic strategy. Scavenging ROS within the mitochondria may protect the heart against the development of heart failure and make it more resistant to stressful stimuli [56]. Our previous studies with *Boerhaavia diffusa* have demonstrated the antioxidant and antihypertrophic potential in H9c2 cells [16], [21]. BDE contains various bioactive phenolic compounds that are potent antioxidants and plays a significant role in the management of diseases associated with oxidative stress. In our study, total phenolic content (TPC) of the BDE was estimated to be 123.76±3.43 mg gallic acid equivalents/g extract and total flavonoid content (TFC) was estimated to be 62·51± 3.19 mg catechin equivalents/g extract. Various active compounds in *B. diffusa* include punarnavine, ursolic acid, punarnavoside, liriodendrin, eupalitin, eupalitin-3-O-â-D-galactopyranoside, rotenoids like boeravinones A, B, C, D, E, F and G, quercetin, kaempferol, etc. [21], [57]. Among these, quercetin exhibits antioxidant, antihypertrophic and antihypertensive potential in *in vitro* and *in vivo* experimental models [58], [59]. Ursolic acid is reported to possess cardioprotective potential via inducing uncoupling of mitochondrial oxidative phosphorylation and reducing mitochondrial H_2_O_2_ production [60]. Eupalitin-3-O-â-D-galactopyranoside is reported to possess immunosuppressive properties and it inhibits the nuclear translocation of NF-êB [61]. Kaempferol is also reported to possess cardioprotective potential and boeravinone G is another antioxidant and genoprotective compound in *B.diffusa* [62], [63]. Liriodendrin isolated from *B.diffusa* is reported to possess Ca^2+^ channel antagonistic properties in heart [64]. Presence of these active constituents might be responsible for its protective activity against Ang II induced hypertrophy.

Overall results reveal that angiotensin II induces alterations in mitochondrial function in H9c2 cells and BDE protects the mitochondria from the deleterious effects of angiotensin II by reducing ROS levels, dissipation of transmembrane potential, opening of mitochondrial permeability transition pore, mitochondrial swelling and enhancing the activities of mitochondrial electron transport chain complexes, aconitase, thioredoxin reductase and also maintained calcium homeostasis through its phenolic mediated antioxidant potential. The outcome of this study shows the possibilities of nutraceuticals from this edible medicinal plant, *Boerhaavia diffusa* for cardiovascular diseases which is a major health issue of the present century. However, further detailed studies are required to establish its molecular mechanisms and therapeutic potential for the maximum utilization of this green leafy vegetable.
